# Building a family at advanced parental age: a systematic review on the risks and opportunities for parents and their offspring

**DOI:** 10.1093/hropen/hoad042

**Published:** 2023-11-15

**Authors:** Nathalie B Neeser, Andrea Martani, Eva De Clercq, Christian De Geyter, Nicolas Vulliemoz, Bernice S Elger, Tenzin Wangmo

**Affiliations:** Institute for Biomedical Ethics, University of Basel, Basel, Switzerland; Institute for Biomedical Ethics, University of Basel, Basel, Switzerland; Institute for Biomedical Ethics, University of Basel, Basel, Switzerland; Reproductive Medicine and Gynaecological Endocrinology (RME), University Hospital, University of Basel, Basel, Switzerland; Centre de Procréation Médicalement Assistée (CPMA), Lausanne, Switzerland; Institute for Biomedical Ethics, University of Basel, Basel, Switzerland; Institute for Biomedical Ethics, University of Basel, Basel, Switzerland

**Keywords:** family building, advanced parental age, advanced maternal age, advanced paternal age, psychosocial wellbeing, mental health, family functioning, medically assisted reproduction

## Abstract

**STUDY QUESTION:**

What is the existing empirical literature on the psychosocial health and wellbeing of the parents and offspring born at an advanced parental age (APA), defined as 40 years onwards?

**SUMMARY ANSWER:**

Although the studies show discrepancies in defining who is an APA parent and an imbalance in the empirical evidence for offspring, mothers, and fathers, there is a drive towards finding psychotic disorders and (neuro-)developmental disorders among the offspring; overall, the observed advantages and disadvantages are difficult to compare.

**WHAT IS KNOWN ALREADY:**

In many societies, children are born to parents at advanced ages and there is rising attention in the literature towards the consequences of this trend.

**STUDY DESIGN, SIZE, DURATION:**

The systematic search was conducted in six electronic databases (PubMed including Medline, Embase, Scopus, PsycInfo, CINAHL, and SocINDEX) and was limited to papers published between 2000 and 2021 and to English-language articles. Search terms used across all six electronic databases were: (‘advanced parental age’ OR ‘advanced maternal age’ OR ‘advanced paternal age’ OR ‘advanced reproductive age’ OR ‘late parent*’ OR ‘late motherhood’ OR ‘late fatherhood’) AND (‘IVF’ OR ‘in vitro fertilization’ OR ‘in-vitro-fertilization’ OR ‘fertilization in vitro’ OR ‘ICSI’ OR ‘intracytoplasmic sperm injection’ OR ‘reproductive techn*’ OR ‘assisted reproductive technolog*’ OR ‘assisted reproduction’ OR ‘assisted conception’ OR ‘reproduction’ OR ‘conception’ OR ‘birth*’ OR ‘pregnan*’) AND (‘wellbeing’ OR ‘well-being’ OR ‘psycho-social’ OR ‘social’ OR ‘ethical’ OR ‘right to reproduce’ OR ‘justice’ OR ‘family functioning’ OR ‘parental competenc*’ OR ‘ageism’ OR ‘reproductive autonomy’ OR ‘outcome’ OR ‘risk*’ OR ‘benefit*’).

**PARTICIPANTS/MATERIALS, SETTING, METHODS:**

The included papers were empirical studies in English published between 2000 and 2021, where the study either examined the wellbeing and psychosocial health of parents and/or their children, or focused on parental competences of APA parents or on the functioning of families with APA parents. A quality assessment of the identified studies was performed with the QATSDD tool. Additionally, 20% of studies were double-checked at the data extraction and quality assessment stage to avoid bias. The variables sought were: the geographical location, the year of publication, the methodological approach, the definitions of APA used, what study group was at the centre of the research, what research topic was studied, and what advantages and disadvantages of APA were found.

**MAIN RESULTS AND THE ROLE OF CHANCE:**

A total number of 5403 articles were identified, leading to 2543 articles being included for title and abstract screening after removal of duplicates. This resulted in 98 articles included for a full-text reading by four researchers. Ultimately, 69 studies were included in the final sample. The key results concerned four aspects relevant to the research goals. (i) The studies showed discrepancies in defining who is an APA parent. (ii) There was an imbalance in the empirical evidence produced for different participant groups (mothers, fathers, and offspring), with offspring being the most studied study subjects. (iii) The research topics studied underlined the increased risks of neuro-developmental and psychotic disorders among offspring. (iv) The observed advantages and disadvantages were varied and could not be compared, especially for the offspring of APA parents.

**LIMITATIONS, REASONS FOR CAUTION:**

Only English-language studies, published between 2000 and 2021, found in the above-mentioned databases were considered for this review.

**WIDER IMPLICATIONS OF THE FINDINGS:**

More research is necessary to understand the risks and benefits of building a family at an APA for the offspring when they reach adulthood. Furthermore, studies that explore the perspective of older fathers and older parents from non-Western societies would be highly informative.

**STUDY FUNDING/COMPETING INTEREST(S):**

The writing of this manuscript was permitted by financial support provided by the Swiss National Science Foundation (Weave/Lead Agency funding program, grant number 10001AL_197415/1, project title ‘Family Building at Advanced Parental Age: An Interdisciplinary Approach’). The funder had no role in the drafting of this manuscript and the views expressed therein are those of the authors. The authors have no conflicts of interest.

**REGISTRATION NUMBER:**

This systematic review is registered in Prospero: CRD42022304564.

WHAT DOES THIS MEAN FOR PATIENTS? In many societies, there is a tendency for people to become parents later in life compared with previous generations. This leads to more and more parents being of so-called ‘advanced parental age’ (APA), which we define here as parents who were 40 years or older at the time their child was born. This demographic development has led to rising attention in the medical literature, especially towards what this means for children of these parents and the APA parents themselves.

We present our findings based on the analysis of the existing empirical studies on the psychosocial health and wellbeing of APA parents and their children. Our systematic search of empirical studies in six databases showed that it is difficult to compare the findings of the different empirical studies because they use different ages to define an APA parent and that many more studies focused on children than on the mothers and fathers. Nevertheless, the empirical studies indicate that there is high interest in the psychosocial health and wellbeing of children, specifically showing an attentiveness towards psychotic disorders and (neuro)developmental disorders. Overall, there are both advantages and disadvantages in connection to being a child of an APA parent.

## Introduction

Advanced parental age (APA) has become an increasing trend in many Western societies. In the European Union, live births from mothers aged 40–44, 45–49, and 50+ have increased steadily in recent years (Eurostat, 2022). Although most studies have focused on advanced maternal age, the average age at first birth is on the rise in many countries for men as well (Khandwala *et al.*, 2017; Destatis, 2020; Statista, 2022).

The upward trend in age of (first) parenthood has sparked debates on a wide range of issues. These include socio-cultural and economic factors (e.g. education, career opportunities, stable relationships) and wider socio-demographic impacts of parenthood at advanced age (e.g. lower birth rate, sustainability of welfare systems) (Kramer, 2014; Cutas and Smajdor, 2015). There are also discussions surrounding the use of medically assisted reproduction (MAR) to address age-related infertility (Zwart, 1994; Adrian *et al.*, 2021). These discussions include reproductive autonomy and cross-border fertility care (Ekberg, 2014; Cutas and Smajdor, 2015; Nahman, 2018), as well as regulatory challenges of demarking an age cut-off for MAR (Cavaliere and Fletcher, 2022). The age cut-off is a particular challenge given the medical risks for both the mother and the child (e.g. caesarean delivery and gestational hypertension) (Berntsen *et al.*, 2019; Pettersson *et al.*, 2020).

Some of the most controversial issues surrounding parenthood at advanced age are probably those that affect the child’s physical, mental, emotional, and social wellbeing. Indeed, the relationship between APA and child wellbeing is complex insofar as the trade-off between risks and benefits is not easy to make (Goisis, 2015; Zondervan-Zwijnenburg *et al.*, 2020). In medical literature, a large number of studies discuss adverse physical and neuro-developmental outcomes in association with APA. These adverse outcomes range from cardiovascular problems and cancer to autism spectrum disorder (ASD) and schizophrenia (Lampi *et al.*, 2013; D'Onofrio *et al.*, 2014; Carslake *et al.*, 2017). Some papers also express concern about the toll of the caregiving burden and of early parental loss on children’s education, professional life, and interpersonal relationships (Zweifel *et al.*, 2020). At the same time, literature outside of the strict clinical context reports important advantages of APA on the child’s psychosocial wellbeing (Zondervan-Zwijnenburg *et al.*, 2020) and cognitive skills (Barclay and Myrskylä, 2016; Carslake *et al.*, 2017; Myrskylä *et al.*, 2017). The fact that ‘older’ parents have more life experience, are more likely to be financially stable, and feel more ‘ready’ for parenthood might compensate for poorer physical and developmental birth outcomes (Myrskylä *et al.*, 2017). Although there is limited research on the lived experiences of ‘older’ parents, it associates parenthood at advanced age with higher levels of wellbeing (George-Carey *et al.*, 2021).

Some reviews on the impact of APA on family building and medical care in reproductive medicine and in obstetrics have been published. Schmidt *et al.* (2012) focused on the demographic (i.e. smaller families, involuntary childlessness) and medical (i.e. infertility, ectopic pregnancies, stillbirths) consequences of parenthood at an APA. Myrskylä *et al.* (2017) examined mainly psychosocial advantages of advanced maternal age on the wellbeing of mother and child. Other scholars studied the paternal dimension of parenthood at advanced age (Brandt *et al.*, 2019; Couture *et al.*, 2021); the former reviewed clinical reproductive risks associated with advanced paternal age (Brandt *et al.*, 2019), while the latter studied terminological, social, public health, psychological, ethical, and regulatory aspects of becoming a father at a later age (Couture *et al.*, 2021). Additionally, a recent mini-review by Lysons and Jadva (2023) highlighted the psychosocial outcomes in connection to APA in early and middle childhood by examining the psychological wellbeing of APA parents and the parent–child relationship. These reviews mostly, but not exclusively, in the case of Lysons and Jadva (2023), highlight the somatic health consequences of APA, consider either fathers or mothers, and focus on the phenomenon of older parenthood rather than a specific age group.

In this review, we map and synthesize the existing empirical research on parents of a specifically defined APA (40 years and onwards) and their offspring, focusing on the psychosocial wellbeing of parents and children, family functioning, and parental competences. We use the term ‘psychosocial’ wellbeing to generally refer to all issues that impact wellbeing, and that do not strictly concern somatic health (e.g. higher risk of cancer for children of APA parents, higher risk of hypertension for APA mothers, etc.). Also, the terms ‘parenthood at advanced age’ and ‘having a child at advanced age’ are used interchangeably, as we see them as two sides of the same coin and do, therefore, not make a specific distinction between social and biological age in the context of this review. Whilst much attention has been invested into the study of the perinatal somatic health of older parents and their children (Lean *et al.*, 2017; Brandt *et al.*, 2019; Pinheiro *et al.*, 2019), we are not aware of a systematic review that summarizes the recent empirical literature on the long-term and psychosocial advantages or disadvantages of having children at an older age. For this reason, we specifically evaluate the empirical evidence of psychosocial wellbeing, family functioning, and parental competences. Also, we identified evidence that describes the consequences of APA across ages (Brown *et al.*, 2002; Durkin *et al.*, 2008; Carslake *et al.*, 2019; Cantalini *et al.*, 2020), but there is no exclusive focus on the ‘oldest’ group of parents. We included studies in which the participating parents were at least 40 years old at the time their offspring was born. While many medical studies define advanced maternal age as 35 years and older (Gantt *et al.*, 2022 on behalf of the Committee on Clinical Consensus-Obstetrics), and advanced paternal age as 40 years and older (Toriello and Meck, 2008), the understanding and definition of the term APA is unclear. We selected ≥40 as a cut-off for this review because we concentrated on both mothers and fathers and captured research on the ‘older’ segments of parents, defined from not just a medical, but also a societal, perspective.

## Materials and methods

The protocol for this systematic review is registered in Prospero and can be accessed through its registration number CRD42022304564. The review follows the Preferred Reporting Items for Systematic Reviews and Meta-Analysis (PRISMA) guidelines (Moher *et al.*, 2009). In January 2022, we conducted a systematic search in six electronic databases: PubMed including Medline, Embase, Scopus, PsycInfo, CINAHL, and SocINDEX. The search was limited to only include empirical studies published between 1 January 2000 and 31 December 2021.

We designed the search strategy based on a population context outcome (PCO) structure (see Table 1), an adaptation of the traditional population intervention comparison outcome scheme to fit the review’s specific aims. Within each component of the PCO, every term was linked to others by Boolean ORs, whereas the linkage across PCO components has been with Boolean ANDs. The search terms included can be found in Table 1. In Supplementary Tables S1, S2, S3, S4, S5, and S6, we present the full electronic search strategy for each database depicting how the search terms were adapted based on the database thesaurus, e.g. MeSH for PubMed, as well as specifications that were possible within different databases. The final search was run on 13 January 2022 and was not re-run prior to the final analysis.

**Table 1.** Search strategy and terms.

PCO structure	Definition	Search terms
Population	Becoming parents at 40 years or older	(“advanced parental age”) OR (“advanced maternal age”) OR (“advanced paternal age”) OR (“advanced reproductive age”) OR (“late parent*”) OR (“late motherhood”) OR (“late fatherhood”)

Context	Becoming parents either naturally or through MAR	(“IVF”) OR (“in vitro fertilization”) OR (“in-vitro fertilization”) OR (“fertilization in vitro”) OR (“ICSI”) OR (“intracytoplasmic sperm injection”) OR (“reproductive techn*”) OR (“assisted reproductive technolog*”) OR (“assisted reproduction”) OR (“assisted conception”) OR (“reproduction”) OR (“conception”) OR (“birth*”) OR (“pregnan*”)

Outcome	Outcomes of being parent(s) at an advanced age either naturally or through MAR or of having parents at an advanced age	(“wellbeing”) OR (“well-being”) OR (“psycho-social”) OR (“social”) OR (“ethical”) OR (“right to reproduce”) OR (“justice”) OR (“family functioning”) OR (“parental competenc*”) OR (“ageism”) OR (“reproductive autonomy”) OR (“outcome*”) OR (“risk*”) OR (“benefit*”)

MAR, medically assisted reproduction.

After duplicate removal using Endnote, two researchers independently screened and assessed the remaining titles and abstracts for their relevance to the research goal. That is, they evaluated whether the papers met the inclusion criteria: (i) empirical studies in English published between 1 January 2000 and 12 December 2021 and (ii) study examining either the wellbeing and psychosocial health of parents and/or their children, focusing on parental competences of APA parents or on the family functioning of families with APA parents. During this process, the two researchers separately decided that (i) the article should be included for full-text screening (i.e. empirical research articles in peer-reviewed journals), or (ii) the article should be excluded as it was not relevant to the research interest (e.g. the article was a theoretical paper, solely focused on physical health indicators, was a different publication to a research article (e.g. a report, a PhD thesis, etc.) or the study did not include ‘older’ parents as defined in this review), or (iii) they were not entirely sure if it was fitting to be included or excluded. All doubtful articles were then discussed between the two researchers, uncertainties were clarified, and unanimous decisions were reached on which articles to include or to exclude. Additional articles were retrieved by citation tracking and scanning references of included papers. The articles deemed relevant for the review were then included for full-text screening.

We carried out a quality assessment of the studies that were included after the full-text screening. For this purpose, we used the QATSDD quality assessment tool, which has been specifically developed for systematic reviews of health-related research, where various study designs are included in the sample (Sirriyeh *et al.*, 2011). The threshold used to determine the methodological soundness of each study was set at 60% to capture data from studies of medium to high quality. In case of remaining doubts about the quality appraisal, the study in question was discussed among the team members and a joint assessment decision was taken. To ensure that the articles were assessed correctly and equally by all three researchers, 20% of the selected articles were independently assessed for their quality of data extraction by a fourth researcher.

A data extraction form was created, so that, for each included study, we systematically collected the following information: general details on the article, such as author(s), title, year, location, and type of research (e.g. qualitative, quantitative, mixed methods); methodological information, such as study design and data collection tools, study objective(s), definition/understanding of APA, sample; and the results of the study, especially including evidence produced on the advantages and disadvantages of APA in terms of psychosocial wellbeing for parents and children, family functioning and parental competences.

We then conducted a narrative synthesis of the extracted data (Popay *et al.*, 2006). To do so, the studies were first mapped for (i) the geographical location they were conducted in, (ii) the year in which they were published, (iii) the methodological approach used, (iv) what definitions of APA authors used, (v) what study group (e.g. only mothers, only fathers, only offspring, the family as a whole) was focused on, (vi) what research topic was studied, such as psychotic disorders, (neuro-)developmental disorders, psychosocial issues, cognitive performance, etc.), and (vii) which advantages and disadvantages of APA came to light and were discussed. The advantages and disadvantages of APA were addressed and grouped according to whom they concern: offspring or parents (either both or only the mother) and themes were created and grouped according to the advantage or disadvantage, such as (in the case for both parents) the family functioning or the psychosocial wellbeing.

## Results

**Figure 1.**
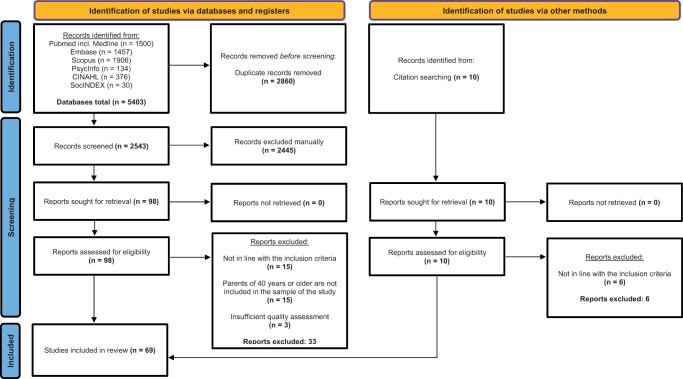
**PRISMA flowchart of included and excluded studies**.

From the six databases stated above, a total number of 5403 articles were identified. Upon removal of duplicates, 2543 articles were screened for title and abstract, resulting in 98 articles included for a full-text reading (see Fig. 1). Thereafter, 33 articles were excluded because either (i) they were not in line with the inclusion criteria (e.g. not an empirical article or relevant to the research goal), (ii) there was uncertainty or (in the case where it was clearly stated) certainty that parents of 40 years or older were not included in the sample of the study, or (iii) the quality assessment was insufficient. Simultaneously, citation searching retrieved 10 additional articles, which were assessed in the same way; of these, only four were in line with the defined inclusion criteria. There were therefore 69 articles included in the final sample of the present systematic review. In the following, we present the findings of our systematic review based on the seven aspects used to analyse the data.

### General features of the included studies

**Figure 2.**
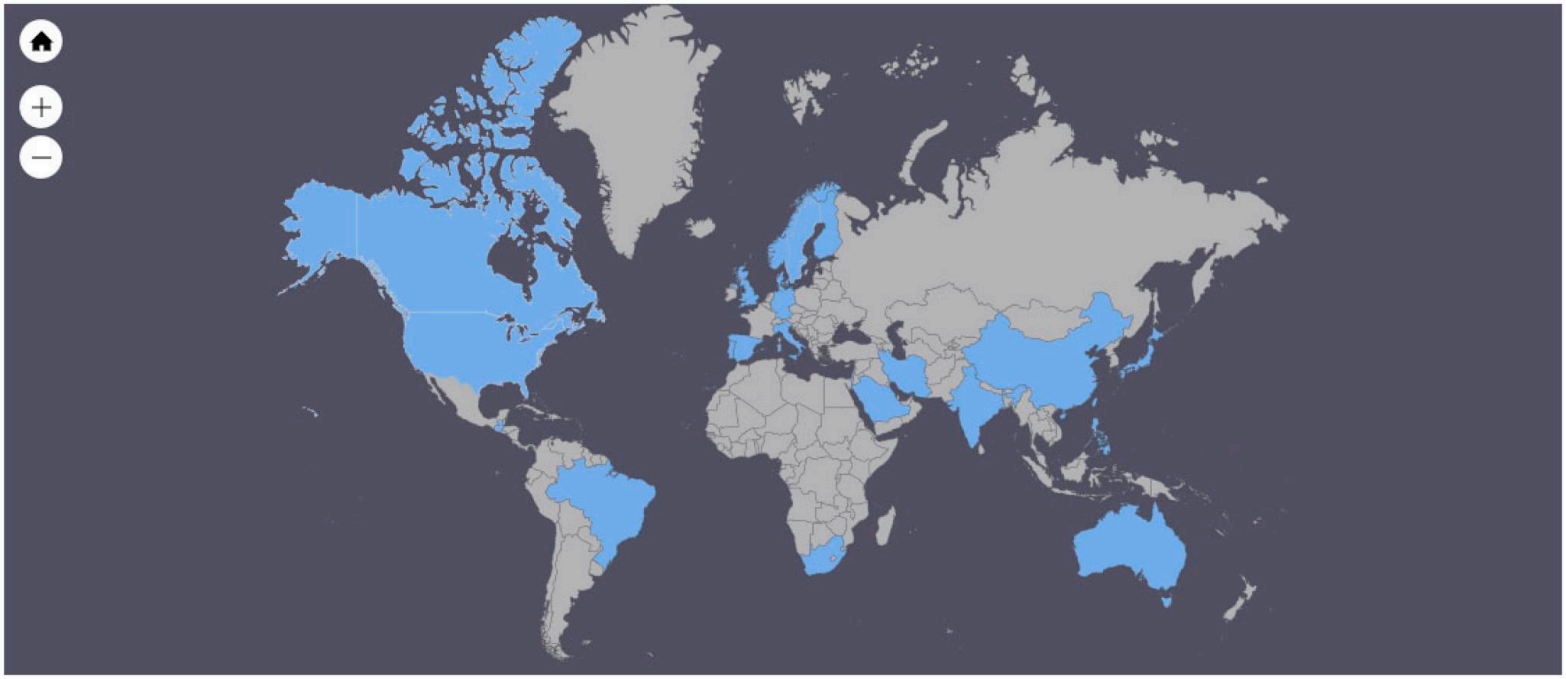
**Overview of countries in which the included studies were implemented**.

The majority (n = 33) of the included studies were carried out in Europe, followed by 24 studies from North America, 19 from Asia, 5 from Australia, 2 from South America, and 1 from Africa (with studies that compare data from different countries and/or continents being listed by country and therefore included multiple times) (Fig. 2). The majority of the studies were conducted in one country alone, but a total of eight studies were conducted in multiple countries: (i) Aasheim *et al.* (2012) in Sweden and Norway; (ii) Byrne *et al.* (2003) in Denmark and the US; (iii) Fall *et al.* (2015) in Brazil, Guatemala, India, the Philippines, and South Africa; (iv) Frans *et al.* (2008) in Sweden and the UK; (v) Frans *et al.* (2011) in Sweden, Australia, the UK, and the US; (vi) Gajos and Beaver (2017) in the US and Saudi-Arabia; (vii) Taylor *et al.* (2019) in the US, Denmark, Germany and Australia; and (viii) Tearne *et al.* (2015) in Australia and Germany.

**Figure 3.**
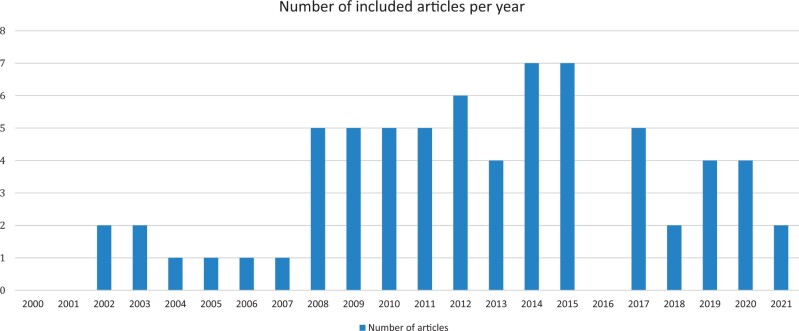
**Number of included articles per year of publication**.

The years in which the included studies were published were evenly distributed from 2002 to 2007 with one or two studies per year. There was a more prominent uprise in publication numbers from 2008 to 2020 with four to eight articles published per year, except in 2016 and 2018 (Fig. 3).

The majority of the included studies (N = 59, 85%) were conducted with quantitative research methods, most being cohort-based studies, followed by survey-based studies and case–control studies (Table 2). Seven studies were qualitative, namely interview-based. Only three employed mixed methods.

**Table 2.** Study characteristics of publications included in this review (n = 69).

Author(s) and year of publication	Location	Study design	Participants	Sample size	Topic(s) of research	Definition of advanced parental age
Aasheim *et al.* (2012)	Sweden and Norway	Longitudinal cohort study	Nulliparous women	19 291 participants	Psychological distress	Mothers (explicit cut-off): ≥32 Fathers: N/A

Ben Itzchak *et al.* (2011)	Israel	Cohort study	Offspring	529 participants diagnosed with autism spectrum disorder and 108 participants with other diagnoses, such as development delay, language disorder, attention deficit hyperactivity disorder	Autism spectrum disorder	Mothers (explicit cut-off): ≥45 Fathers (explicit cut-off): ≥40

Boivin *et al.* (2009)	UK	Survey study	Both parents and offspring	642 participants	Psychological wellbeing	Mothers (explicit cut-off): ≥38 Fathers: N/A

Brown *et al.* (2002)	USA	Cohort study	Offspring	68 participants diagnosed with schizophrenia and 7641 control group participants	Schizophrenia	Mothers: N/A Fathers (stratification): ≥45

Brown *et al.* (2013)	USA	Nested control study	Offspring	94 participants diagnosed with bipolar disorder and 746 control group participants	Bipolar disorder	Mothers (stratification): ≥40 Fathers (stratification): ≥45

Byrne *et al.* (2003)	Denmark and USA	Longitudinal nested case–control study	Offspring	7,704 participants diagnosed with ICD-8 and ICD-10 schizophrenia and 192 590 control group participants	Schizophrenia	Mothers (stratification): ≥40 Fathers (stratification): ≥50

Cantalini *et al.* (2020)	Italy	Cohort study	Offspring (aged 15–18)	213 538 participants	Cognitive performance	Mothers (explicit cut-off): ≥40 Fathers (explicit cut-off): ≥45

Carolan (2003)	Australia	Longitudinal qualitative interview study	Primiparae women aged >35	20 participants	General experience(s)	Mothers (explicit cut-off): ≥35 Fathers: N/A

Carolan (2005)	Australia	Longitudinal qualitative interview study	Mothers	22 participants	General experience(s)	Mothers (explicit cut-off): ≥35 Fathers: N/A

Carslake *et al.* (2019)	UK	Cohort study	Offspring	5 204 433 participants	Mortality	Mothers (stratification): ≥40 Fathers (stratification): ≥45

Chen and Landau (2015)	Israel	Qualitative interview study	Women	20 participants	General experience(s)	Mothers (explicit cut-off): ≥45 Fathers: N/A

Dalman and Allebeck (2002)	Sweden	Population-based case–control study	Offspring	524 participants diagnosed with schizophrenia and 1,043 control group participants	Schizophrenia	Mothers: N/A Fathers (stratification): ≥45

Durkin *et al.* (2008)	UK	Case-cohort study	Offspring (aged 8 years)	1251 participants	Autism spectrum disorder	Mothers (explicit cut-off): ≥35 Fathers (explicit cut-off): ≥40

Engelhardt and Schreyer (2014)	Germany	Longitudinal survey study	Parents (aged 50–79)	6623 participants	General experience(s)	Mothers (explicit cut-offs): 28–42 (1952–1961); 26–43 (1942–1951); 27–48 (1932–1948). Fathers (explicit cut-offs): 31–51 (1952–1961); 32–54 (1942–1951); 31–58 (1932–1948).
Fall *et al.* (2015)	Brazil, Guatemala, India, Philippines, South Africa	Longitudinal cohort study	Mothers and their offspring	19 403 participants and their children	Cognitive performance	Mothers (explicit cut-off): ≥35 Fathers: N/A

Frans *et al.* (2008)	Sweden and UK	Nested case–control study	Offspring	13 428 participants diagnosed with bipolar disorder and 67 140 control group participants	Bipolar disorder	Mothers (explicit cut-off): ≥45 Fathers (explicit cut-off): ≥55

Frans *et al.* (2011)	Sweden, Australia, UK, USA	Multi-generational case–control study	Offspring	2511 participants diagnosed with schizophrenia and 15 619 control group participants	Schizophrenia	Mothers (explicit cut-off): ≥45 Fathers (explicit cut-off): ≥55

Friese *et al.* (2008)	USA	Qualitative interview study	Couples	79 couples	General experience(s)	The authors do not offer a definition of APA.

Gajos and Beaver (2017)	USA and Saudi Arabia	Longitudinal mixed-method study (Cohort and interview study)	Families	929 families	Cognitive performance	The authors do not offer a definition of APA.

George-Carey *et al.* (2021)	UK	Survey study	Mothers	13 participants	General experience(s)	Mothers (explicit cut-off): ≥50 Fathers: N/A

Ghanizadeh (2014)	Iran	Cross-sectional study	Offspring and their parents	470 participants	Attention deficit hyperactivity disorder	The authors do not offer a definition of APA.

Goisis (2015)	UK	Retrospective, cohort-based demographic study	Mothers and their offspring	5257 participants	General experience(s)	Mothers (stratification): ≥40 Fathers: N/A

Guedes and Canavarro (2014)	Portugal	Longitudinal survey study	First-time parents	58 couples (mothers ≥35) and 41 control group couples (mothers 20–34 years old)	Psychological adjustment	Mothers (explicit cut-off): ≥35 Fathers: N/A

Guedes and Canavarro (2015)	Portugal	Longitudinal survey study	First-time parents	74 couples (mothers ≥35) and 71 control group couples (mothers 20–34 years old)	Psychological adjustment	Mothers (explicit cut-off): ≥35 Fathers: N/A

Hui *et al.* (2015)	Hong Kong	Prospective survey-based study	Offspring	191 participants diagnosed with first-episode-psychosis	Schizophrenia	Mothers: N/A Fathers (stratification): ≥40

Hultman *et al.* (2011)	Sweden	Epidemiological registry-based study	Offspring	660 participants diagnosed with autism spectrum disorder and their non-affected siblings	Autism spectrum disorder	Mothers (stratification): ≥40 Fathers (stratification): ≥50

Janecka *et al.* (2017a)	UK	Cohort study	Offspring (twins)	8601 participants (4528 families)	Cognitive performance	Mothers (stratification): ≥50 Fathers (stratification): ≥50

Janecka *et al.* (2017b)	UK	Population-based cohort study	Offspring (twins)	22 322 participants	Behavioural issues	Mothers (stratification): ≥40 Fathers (stratification): ≥50

Javaras *et al.* (2017)	Sweden	Register-based cohort study	Offspring	2 276 809 participants	Eating disorders	Mothers (stratification): ≥45 Fathers: (stratification): ≥45
King *et al.* (2009)	USA	Register-based cohort study	Offspring	18 731 participants diagnosed with autism spectrum disorder and 4 888 195 control group participants	Autism spectrum disorder	Mothers (explicit cut-off): ≥40 Fathers (explicit cut-off): ≥40

Lampi *et al.* (2013)	Finland	Retrospective epidemiological study, both register and cohort based	Offspring	4713 participants diagnosed with autism spectrum disorder and 18 777 control group participants	Autism spectrum disorder	Mothers (stratification): ≥40 Fathers (stratification): ≥50

Lan *et al.* (2021)	Taiwan	Retrospective epidemiological study, both register and cohort based	Offspring	17 649 participants diagnosed with schizophrenia and 70 596 control group participants	Schizophrenia	Mothers (stratification): ≥50 Fathers (stratification): ≥50

Lopez-Castroman *et al.* (2010)	Spain	Retrospective clinical and population-based epidemiological study	Offspring (until age 18)	30 965 participants	Schizophrenia	Mothers (stratification): ≥50 Fathers (stratification): ≥50

Lung *et al.* (2018)	Taiwan	Retrospective cohort study	Offspring	20 095 participants	Autism spectrum disorder	Mothers (explicit cut-off): ≥40 Fathers (explicit cut-off): ≥50

Mac Dougall *et al.* (2012)	USA	In-depth qualitative interviews	Parents (mothers aged 40 or older at time of delivery of their first child)	46 couples and 15 individuals	General experience(s)	Mothers (explicit cut-off): ≥40 Fathers: N/A

Mandel (2010)	USA	Semistructured qualitative interviews	Single mothers (aged 35–48)	11 participants	General experience(s)	Mothers (explicit cut-off): ≥35 Fathers: N/A

Manzouri *et al.* (2019)	Iran	Cross-sectional study	Offspring (aged 16–30 months) and their mothers	1504 mother–child pairs	Autism spectrum disorder	Mothers (explicit cut-off): ≥35 Fathers (explicit cut-off): ≥40

Merikangas *et al.* (2017)	USA	Cohort study	Offspring (aged 8–21 years)	8725 participants	Psychological wellbeing	Mothers (stratification): ≥40 Fathers (stratification): ≥45

Meyer (2020)	USA	Survey-based study	First-time mothers (up to age 5)	147 participants	Psychological needs	Mothers (explicit cut-off): ≥40 Fathers: N/A

Miller *et al.* (2010a)	Finland	Longitudinal cohort study	Offspring	10 965 participants	Mortality	Mothers (stratification): ≥45 Fathers (stratification): ≥45

Miller *et al.* (2010b)	Finland	Longitudinal cohort study	Offspring (followed from age 46–55)	529 participants diagnosed with nonaffective psychosis disorders	Mortality	Mothers (stratification): ≥40 Fathers (stratification): ≥40

Miller *et al.* (2011)	Finland	Cohort study	Adult offspring and their parents	13 712 participants diagnosed with nonaffective psychosis and 10 224 control group participants	Schizophrenia	Mothers (stratification): ≥50 Fathers (stratification): ≥50

Muraca and Joseph (2014)	Canada	Population-based study	Mothers (aged 20–44)	7936 participants	Depression	Mothers (explicit cut-off): ≥35 Fathers: N/A

Myrskylä *et al.* (2013)	Sweden	Cohort study	Offspring and their siblings	565 433 participants	Cognitive performance	Mothers (stratification): ≥45 Fathers (stratification): ≥50
Nelson (2004)	USA	Qualitative interviews combined with literature review and findings from other studies	First-time mothers (aged 36–48)	7 participants	General experience(s)	Mothers (explicit cut-off): ≥35 Fathers: N/A

Nilsen *et al.* (2012)	Norway	Cross-sectional cohort study	Nulliparous women	41 236 participants	General experience(s)	Mothers (explicit cut-off): ≥38 Fathers: N/A

Opler *et al.* (2013)	USA	Post hoc examination of data from double-blind, placebo-controlled study	Offspring (aged 12–17 years)	288 participants	Schizophrenia	The authors do not offer a definition of APA.

Petersen *et al.* (2011)	Denmark	Cohort study	Offspring (aged until 15 years)	2 200 000 participants	Schizophrenia	Mothers (stratification): ≥40 Fathers (stratification): ≥55

Racine (2014)	USA	Population-based cohort study	Offspring (female twins)	1722 participants	Eating disorders	Mothers: N/A Fathers (stratification): ≥40

Reichenberg *et al.* (2006)	Israel	Historical population-based cohort study	Offspring	378 891 participants	Autism spectrum disorder	Mothers: N/A Fathers (stratification): ≥50

Rieske and Matson (2020)	USA	Clinical cohort study	Offspring (aged 2–17 years)	252 participants	Autism spectrum disorder	The authors do not offer a definition of APA

Saha *et al.* (2009a)	USA	Cohort study	Offspring (until age 7)	55 908 participants	Behavioural issues	Mothers (stratification): ≥40 Fathers (stratification): ≥40

Saha *et al.* (2009b)	USA	Cohort study	Offspring	33 437 participants	Cognitive performance	Mothers: N/A Fathers (stratification): ≥40

Shelton and Tancredi (2010)	USA	Retrospective population-based cohort study	Offspring (until age 6)	12 159 participants and 4 935 776 control group participants	Autism spectrum disorder	Mothers (stratification): ≥40 Fathers (stratification): ≥40

Shimada *et al.* (2012)	Japan	Retrospective chart review study	Offspring	762 participants diagnosed with autism spectrum disorder, attention deficit hyperactivity disorder, or tourette syndrome	Autism spectrum disorder	Mothers (stratification): ≥40 Fathers (stratification): ≥40

Sørensen *et al.* (2014)	Denmark	Cohort study	Male offspring (male)	138 966 participants	Schizophrenia	Mothers (stratification): ≥45 Fathers (stratification): ≥35

Steiner and Paulson (2007)	USA	Prospective cohort study	Mothers	18 participants who conceived and delivered after age 50; 24 participants in their 40s and 22 participants in their 30s	Psychological distress	Mothers (explicit cut-off): ≥50 Fathers: N/A

Taylor *et al.* (2019)	USA, Denmark, Germany, Australia	Risk analysis study	Offspring	13 436 participants diagnosed with intellectual disability, neuro-developmental disorders, congenital heart disease, autism spectrum disorder, schizophrenia and 3813 control group participants	Psychological wellbeing	Mothers: N/A Fathers (explicit cut-off): ≥40
Tearne *et al.* (2015)	Australia, Germany	Longitudinal cohort study	Offspring	1754 participants	Behavioural issues	Mothers (stratification): ≥40 Fathers (stratification): ≥40

Torrey *et al.* (2009)	USA	Cohort study	Offspring	168 participants	Schizophrenia	Mothers: N/A Fathers (stratification): ≥55

Tsuang *et al.* (2014)	USA	Prospective quantitative study	Offspring	293 participants diagnosed with schizophrenia and 382 of their non-affected siblings	Schizophrenia	Mothers: N/A Fathers (explicit cut-off): ≥40

Tsuchiya *et al.* (2008)	Japan	Prospective quantitative study including retrospective analysis	Offspring	84 participants diagnosed with high functioning Autism spectrum disorder and 208 control group participants	Autism spectrum disorder	Mothers (explicit cut-off): ≥31 Fathers (explicit cut-off): ≥35

Van Balkom *et al.* (2012)	Aruba	Retrospective case–control, population-based study	Offspring	101 participants diagnosed with autism spectrum disorder and 469 control group participants	Autism spectrum disorder	Mothers (stratification): ≥40 Fathers (stratification): ≥50

Wang *et al.* (2015)	Taiwan	Prospective survey study	Offspring	1297 participants	Schizophrenia	Mothers (stratification): ≥40 Fathers (stratification): ≥50

Wang *et al.* (2019)	Taiwan	Mixed method quantitative study (including genetic data)	Offspring	2923 participants diagnosed with schizophrenia	Schizophrenia	Mothers (stratification): ≥35 Fathers (stratification): ≥45

Weiser *et al.* (2008)	Israel	Retrospective registry-based study	Offspring (aged 16–17)	403 565 participants	Schizophrenia, autism spectrum disorder	Mothers (stratification): ≥40 Fathers (stratification): ≥45

Weiser *et al.* (2020)	Israel	Retrospective registry-based study	Offspring (aged 16–17)	911 951 participants	Schizophrenia, bipolar disorder	Mothers (stratification): ≥40 Fathers (stratification): ≥45

Wen *et al.* (2018)	Taiwan	Quantitative survey-based study	Mothers	160 participants	Sleep quality	Mothers (explicit cut-off): ≥35 Fathers: N/A

Wu *et al.* (2012)	China	Quantitative survey-based study	Offspring (male and female)	351 participants diagnosed with schizophrenia; 122 participants diagnosed with obsessive–compulsive disorder; and 238 control group participants	Schizophrenia	Mothers (stratification): ≥35 Fathers (stratification): ≥35

APA, advanced parental age; N/A, not applicable; ICD, International Statistical Classification of Disease and Related Health Problems.

### The definition of APA

Across the studies, there was no shared understanding of the definition of APA, nor of the chronological cut-off to determine which parents can be ascribed to the APA category (for exact definitions, see Table 2). In the following, we focus on the three different ways in which APA was defined. While some papers (n = 30) provided a specific age to describe parents of APA, others (n = 34) did not provide explicit age boundaries, but rather presented findings based on categorical age groups. A few papers (n = 5) treated parental age as a continuous variable (rather than a categorical variable, e.g. every parent above 40 years of age) and no specific age cut-off was used to demarcate APA in these few papers. An example of the use of continuous variables is Rieske and Matson (2020) who did not set a specific chronological cut-off to define at what age a parent is categorized as being of APA, but who rather reflected on the topic of APA and its relationship to the severity of autism symptoms.

**Figure 4.**
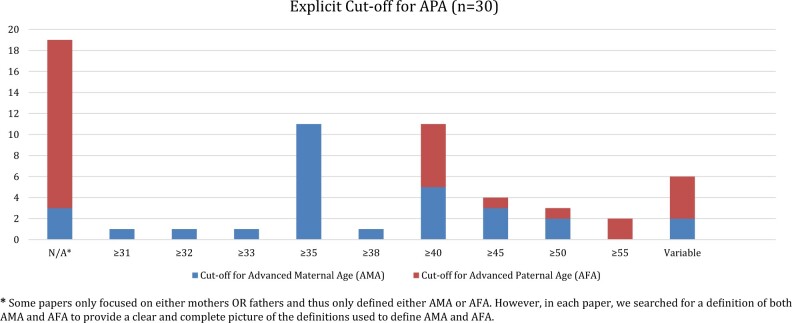
**Defined cut-offs for advanced paternal age in the included studies**.

In the majority (n = 27) of the 30 papers that used an explicit age to define APA, the authors referred to a specific age group within their samples that were categorized as older mothers or fathers but did not reflect on how this age cut-off was determined. The cut-off age used to define when parents can be considered of APA or ‘older’ varied as highlighted in Fig. 4. For mothers, the definition of APA ranged from 31+ to 50+, whereas for fathers it varied between 40+ and 50+. In a few cases, the studies used variable definitions for parents of APA. For example, Engelhardt and Schreyer (2014) adjusted the definition of APA based also on the birth cohort in the sample: for parents born between 1952 and 1961, APA was defined as 31–51 and AMA as 28–42, but for parents born between 1942 and 1951, APA was considered to be 32–54 and AMA as 26–43.

Only in a small proportion of studies with an explicit definition (n = 3), the authors reflected on their definition of APA, and on the reasons why they selected a specific cut-off. In short, they determined the boundaries of parents who belong to this category by a mix of demographic data of the population from which their sample was drawn or on calculations related to the sample itself. Boivin *et al.* (2009) defined AMA at 38 writing that ‘The childbearing period in the reproductive life cycle is generally defined as between the ages of 15 and 44 years in prevalence studies but in reality, fertility is significantly reduced from 38 years onward […]. The age of 38 therefore represents a genuine biological marker of reduced expectation for fertility’ (Boivin *et al.*, 2009:1949f.). Nilsen *et al.* (2012) defined within their sample 33–37 as AMA and 38+ as very AMA. Finally, Durkin *et al.* (2008) explained in their sample that ‘the odds of [the offspring] developing ASD [Autism Spectrum Disorder] […] increased for maternal age ≥35 and paternal age ≥40 years’ and that they ‘therefore used these age cut-offs (maternal age ≥35, paternal age ≥40 years) to classify each parent’s age as “older” versus “younger”’ (Durkin *et al.*, 2008:1270).

**Figure 5.**
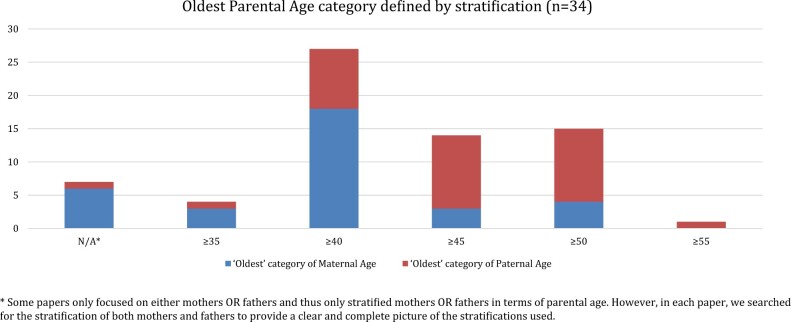
**Oldest parental age category defined by stratification among the included studies.** These studies stratified their samples based on the age of mothers and/or fathers at the time of conception and presented their results based on parental age as a categorical variable.

In 34 papers, there was no definition of a specific cut-off age from which parents can be defined as ‘old’. These studies simply stratified their samples based on the age of mothers and/or fathers at the time of conception and presented their results based on parental age as a categorical variable. There were, nevertheless, significant differences in the stratification of their samples with respect to the ‘oldest’ category of parents they analysed (Fig. 5). Some studies treated all parents aged 35+ within the same categorical variable (e.g. Wu *et al.*, 2012 for both parents, Sørensen *et al.*, 2014 for fathers, and Wang *et al.*, 2019 for mothers), whereas in one paper the category of oldest parents included only those above 55 years of age (Torrey *et al.*, 2009).

The choice of the (oldest) categorical variables in the stratified samples was often taken for granted and it was discussed more reflectively only in a handful of cases. One study explained that ‘maternal and paternal age was considered as both continuous and categorical variables. For the categorical models, maternal and paternal age at birth of the study child was classified within 5-year age categories as per those widely used in population fertility data’ (Tearne *et al.*, 2015:43). Wu *et al.* (2012) revealed that their choice to have 35+ as the oldest category in the stratification of their sample for fathers was that ‘due to the small sample size of fathers aged below 20 or over 40 in both the patient groups and the control group, we set the lowest and highest paternal age category at <25 and ≥35 years old’ (Wu *et al.*, 2012:354). Another paper admitted that the choice to avoid a direct definition of APA for fathers is connected to the fact that ‘in a meta-analysis, we did not find an obvious cut-off point beyond which paternal age should be considered “advanced”’ (Miller *et al.*, 2011:125).

In the five remaining studies, no explicit (i.e. by labelling a specific age from which parents can be considered of APA) or implicit (i.e. by stratification of the sample and identification of the oldest age group) definition of APA was offered. Gajos and Beaver (2017), Opler *et al.* (2013), and Rieske and Matson (2020) treated age as a continuous rather than categorical variable. Whilst their samples included parents above the age of 40, they did not set explicit cut-offs that define APA, but rather reflect on the topic in terms of advancing parental age. One study discussed ‘older motherhood’, but rather than drawing a chronological limit for this category, it spoke of the effect of reproductive technologies allowing women to conceive children also beyond menopause, and thus ‘older mothers can be understood as part of [a] new middle age, engaging in new life course possibilities’ (Friese *et al.*, 2008:66).

### The participants at the centre of APA research

A majority of the studies (n = 43, 62%) focused on the offspring of ‘older’ parents (see Table 2, column ‘Participants’). Most (n = 40) of them did not specify the sex of the offspring in question. Three studies (Weiser *et al.*, 2008, 2020; Myrskylä *et al.*, 2013) focused on male offspring only. A quarter of the included sample (n = 17) were studies that focused on parents: 5 studies on couples (Friese *et al.*, 2008; Mac Dougall *et al.*, 2012; Engelhardt and Schreyer, 2014; Guedes and Canavarro, 2014, 2015) and 12 studies on mothers. None of the studies solely focused on fathers. Finally, nine of the included studies concentrated on the family as a whole, for instance on mothers and their offspring, in the paper of Goisis (2015), or on the family environment and parent and child wellbeing, in the paper of Boivin *et al.* (2009).

### Topics of research

There were three major topics on which research focused (see column ‘Topic(s) of research’ in Table 2 for details).

In 36 studies, the main topic was specific mental disorders in the offspring. Among these 36 studies, 21 addressed psychotic disorders: 16 studies focused solely on schizophrenia, 2 focused exclusively on bipolar disorder, and 3 on multiple aspects of wellbeing such as schizophrenia, bipolar disorder, and ASD. Of the 36 studies, 15 addressed (neuro-)developmental disorders: 12 focused specifically on ASD, 1 on attention deficit hyperactivity disorder (ADHD), and 2 studies on a multitude of aspects such as ASD, ADHD, and Tourette syndrome.

In another 12 studies, the main topic assessed was lived experiences and family wellbeing of the involved participants. Amongst them, the majority described the experiences of first-time mothers. Others focused on experiences of medically assisted reproduction (MAR) in connection to older motherhood (Friese *et al.*, 2008; Boivin *et al.*, 2009; Mac Dougall *et al.*, 2012; Chen and Landau, 2015). Another subgroup of studies addressed aspects influencing the overall wellbeing of family members, such as the timing of first birth (Engelhardt and Schreyer, 2014), becoming a mother over the age of 50 (George-Carey *et al.*, 2021), or pregnancy complications of single women (Mandel, 2010). Finally, one study focused on the overall wellbeing of children of older mothers (Goisis, 2015).

In the remaining studies, the main topic was participants’ wellbeing in relation to psychosocial aspects. More specifically, five studies focused on psychosocial issues, such as psychological distress (Steiner and Paulson, 2007; Aasheim *et al.*, 2012), psychosocial adjustment (Guedes and Canavarro, 2014, 2015), or the psychosocial needs of first-time mothers over 40 (Meyer, 2020). Another six studies addressed deviating cognitive performance of offspring (Saha *et al.*, 2009b; Myrskylä *et al.*, 2013; Fall *et al.*, 2015; Gajos and Beaver, 2017; Janecka *et al.*, 2017a; Cantalini *et al.*, 2020) such as educational outcomes or intelligence that impacted the overall wellbeing of offspring to APA parents. Three studies discussed behavioural issues of the offspring resulting in altered social development (Janecka *et al.*, 2017b) and behavioural dysfunction (Saha *et al.*, 2009a; Tearne *et al.*, 2015). Two studies focused on eating disorders (Racine, 2014; Javaras *et al.*, 2017), one on depression (Muraca and Joseph, 2014), and one on sleep quality (Wen *et al.*, 2018), all influencing the offspring’s overall wellbeing. Three studies stood out in that the topic was mortality (Miller *et al.*, 2010a,b; Carslake *et al.*, 2019).

### Advantages and disadvantages of APA

In the following, we outline and categorize empirical evidence concerning the advantages and disadvantages of being an APA parent or a child to ‘older’ parents. All in all, 57 of the included studies reported at least one disadvantage of having children later in life. Since some studies concluded that there is more than one disadvantage of having children later (e.g. higher risk of ASD and Schizophrenia for the child), the absolute number of times when a disadvantage was mentioned was higher (85 times). Conversely, across a total of 22 studies advantages were mentioned only 40 times. Most disadvantages were identified related to offspring (58 times across 45 studies), while for offspring alone, an advantage was identified only 12 times (across 11 studies). When it comes to parents, most of the identified aspects focused on mothers alone: 22 times, an advantage across nine studies and 22 times, a disadvantage across 12 studies; while in connection to both parents, a positive aspect was identified six times across 2 studies, and negative aspects were identified a total of 5 times in 1 study. No advantages nor disadvantages related to APA fathers alone were discussed. The advantages and disadvantages were grouped into different thematic categories (see Table 3) created by grouping all detected advantages and disadvantages into similar categories. These categories were discussed among three authors and approved by the others.

**Table 3.** Advantages and disadvantages of advanced parental age.

Themes	Advantages of being an offspring of ‘older parents’	Related articles	Disadvantages of being an offspring of ‘older parents’	Related articles
**Psychological aspects**	**Benefits related to psychotic disorders**		**Psychotic disorders**	
	Better response to schizophrenia treatment Decreased risk of schizophrenia relapse Decreased risk of bipolar disorder	Opler *et al.* (2013) Hui *et al.* (2015) Brown *et al.* (2013)	Increased risk of schizophrenia Increased risk of schizophrenia relapse Earlier onset of schizophrenia Increased risk of schizophrenia (based on maternal grandfather’s age) Increased risk of psychotic disorder Increased risk of bipolar disorder Increased risk of hospital admission	Brown *et al.* (2002), Byrne *et al.* (2003), Dalman and Allebeck (2002), Frans *et al.* (2011), Lan *et al.* (2021), Miller *et al.* (2011), Petersen *et al.* (2011), Sørensen *et al.* (2014); Taylor *et al.* (2019); Torrey *et al.* (2009), Tsuang *et al.* (2014), Weiser *et al.* (2020), and Wu *et al.* (2012) Hui *et al.* (2015) Wang *et al.* (2015, 2019) Frans *et al.* (2011) Lopez-Castroman *et al.* (2010) Weiser *et al.* (2020) and Frans *et al.* (2008) Sørensen *et al.* (2014)
			**(Neuro-)developmental disorders**	
			Increased risk of autism spectrum disorder (ASD) Increased risk of attention-deficit hyperactivity disorder (ADHD) Increased risk of ADHD severity Increased risk of Asperger’s syndrome Increased risk of pervasive developmental disorders (PDD)	Ben Itzchak *et al.* (2011), Durkin *et al.* (2008), Hultman *et al.* (2011), King *et al.* (2009), Lampi *et al.* (2013), Lung *et al.* (2018), Manzouri *et al.* (2019), Merikangas *et al.* (2017), Reichenberg *et al.* (2006), Rieske and Matson (2020), Shelton and Tancredi (2010), Shimada *et al.* (2012), Taylor *et al.* (2019), Tsuchiya *et al.* (2008), and Van Balkom *et al.* (2012) Janecka *et al.* (2017b) and Shimada *et al.* (2012) Ghanizadeh (2014) Lampi *et al.* (2013) Lampi *et al.* (2013) and Merikangas *et al.* (2017)
			**Psychosocial issues**	
			Increased anxiety Increased loneliness Fear of losing one’s mother early	Chen and Landau (2015) ^a^ Chen and Landau (2015) Chen and Landau (2015)
**Cognitive aspects**	Higher educational outcomes Increased geek index (GI) Good neurocognitive performance	Cantalini *et al.* (2020) and Fall *et al.* (2015) Janecka (2017a) Saha (2009b)	Inferior neurocognitive performance Decreased verbal IQ scores Lower IQ Increased risk of mental retardation	Saha *et al.* (2009b) Gajos and Beaver (2017) Myrskylä *et al.* (2013) Lopez-Castroman *et al.* (2010)
**Behavioural aspects**	Decrease of risk of bad behaviour	Saha (2009a) and Tearne *et al.* (2015)	Increased risk of bad behaviour Higher risk of poor social functioning Difficulties in emotionality Difficulties in conducting problems	Saha *et al.* (2009a) Weiser *et al.* (2008) Janecka *et al.* (2017b) Janecka *et al.* (2017b)
**Nutritional aspects**	Improved nutritional status	Fall *et al.* (2015)	Increased risk of anorexia nervosa and other eating disorders Higher risk of eating disorders Increased risk of obesity	Javaras *et al.* (2017) Racine (2014) Goisis (2015)
**Life expectancy**	Decreased suicide and all-cause mortality Improved adult survival	Miller (2010a) Carslake *et al.* (2019)	Increased suicide/mortality in female offspring	Miller (2010a,b)

**Table 3.** Continued

Themes	Advantages of being an ‘older parent’	Related articles	Disadvantages of being an ‘older parent’	Related articles
**Psychological aspects**	**Parents in general**		**Parents in general**	
	Increased sense of emotional preparedness Increased awareness of autism spectrum disorder in their offspring	Mac Dougall *et al.* (2012) Durkin *et al.* (2008)	Fear of social stigma	Mac Dougall *et al.* (2012)
	**APA mothers**		**APA mothers**	
	Increased psychosocial wellbeing Increased sense of stability Increased sense of feeling wise Increased confidence and self-assurance Increased satisfaction about motherhood Increased sense of preparedness for parenting Increased appreciation of children Better mental functioning and less stress	George-Carey *et al.* (2021) Carolan (2005), Friese *et al.* (2008), and Meyer (2020) Meyer (2020) and Nelson (2004) Carolan (2005), Friese *et al.* (2008), and Nelson (2004) George-Carey *et al.* (2021) Carolan (2005), Mandel (2010), Meyer (2020), and Nelson (2004) Nelson (2004) Steiner and Paulson (2007)	Increased psychological distress in mothers with a history of depression Increased risk of depression Increased sense of vulnerability and loss of control Fear of social stigma Fear of loss of independence Increased concerns of staying young/healthy Poorer sleep quality In case MAR was used to conceive: insecurity on how to tell child how it was conceived	Aasheim *et al.* (2012) Boivin *et al.* (2009), Carolan (2003), and Muraca and Joseph (2014) Mandel (2010) Chen and Landau (2015), Friese *et al.* (2008), and Meyer (2020) Meyer (2020) and Nelson (2004) Carolan (2005), Friese *et al.* (2008), and Meyer (2020) Wen *et al.* (2018) Mandel (2010)
**Family-functioning**	**Parents in general**		**Parents in general**	
	Stable relationship and functioning co-parenting Positive overall family experience	Mac Dougall *et al.* (2012)	Decreased physical energy Smaller family size Less lifetime with offspring Increased infertility and need to use IVF to conceive	Mac Dougall *et al.* (2012)
			**APA mothers**	
			Perceived lack of energy Difficulty in transition to motherhood Perceived lack of family support Fear of not seeing grandchildren Lower social status and atypical relationship status	Carolan (2005) and Meyer (2020) Carolan (2003) Mandel (2010) and Meyer (2020) Friese *et al.* (2008) Nilsen *et al.* (2012)
**Behavioural aspects**	**APA mothers**			
	Better health behaviour	Boivin *et al.* (2009)		
**Socio-economic aspects**	**Parents in general**			
	Increased financial security Work-time flexibility	Mac Dougall *et al.* (2012)		
	**APA mothers**			
	Increased professional security Increased financial security Better socio-economic position	Aasheim *et al.* (2012) Friese *et al.* (2008), Mandel (2010), and Meyer (2020) Boivin *et al.* (2009)		

APA, advanced parental age; MAR, medically assisted reproduction.

a The study by Chen and Landau (2015) is a qualitative study based on interviews with APA mothers (see Table 2). However, since in their direct results, they also reported how APA mothers felt that their offspring were impacted, we still reported this result here for completeness. The authors state the following: “Participants perceived their children as feeling different from peers because of their mothers’ advanced age and its implications. Children were preoccupied with the possibility of losing their mother early. This, together with not having a sibling, aroused anxiety, yearning and loneliness among the children who were compelled to develop special coping strategies” (2015:29).

#### Advantages to offspring of ‘older parents’

The detected advantages in connection to offspring’s wellbeing (n = 12) were grouped into five categories. The majority of studies identified better cognitive performance (n = 4) (e.g. higher educational outcomes, good neurocognitive performance), followed by benefits related to psychotic disorders (n = 3) (e.g. a decreased risk of bipolar disorder, better response to schizophrenia treatment and a decreased risk of schizophrenia relapse). The remaining three categories included advantages related to behavioural issues (n = 2), life expectancy (n = 2), and nutritional aspects (n = 1).

#### Disadvantages to offspring of ‘older parents’

As for the disadvantages detected in the empirical research findings (n = 58), the majority (n = 21) were detected in connection to (neuro-)developmental disorders (e.g. an increased risk of ASD, an increased risk of ADHD and ADHD severity, Asperger’s syndrome and pervasive developmental disorders). This category was followed by evidence presenting disadvantages in connection to psychotic disorders (n = 21) (e.g. increased risk of schizophrenia, increased risk of schizophrenia relapse and its earlier onset, increased risk of bipolar disorder). The less-mentioned categories included cognitive aspects (n = 4), behavioural aspects (n = 4), psychosocial issues (n = 3), nutritional aspects (n = 3), and life expectancy (n = 2).

#### Advantages to the ‘older parent’

As for the empirical evidence indicating advantages for both parents, we found two in connection to psychological aspects (e.g. increased sense of emotional preparedness), socio-economic aspects (e.g. increased financial security and work-time flexibility), and family functioning (e.g. positive overall family experience, stable relationship, and functioning co-parenting) (see Table 3). None of the empirical findings of the included studies focused on advantages of fathers alone. In the evidence highlighting benefits to APA mothers alone, 22 advantages were mentioned in connection to psychological aspects (n = 16) (e.g. increased psychosocial wellbeing, increased sense of stability, increased sense of feeling wise, self-assured and confident, increased sense of preparedness for parenting, appreciation of their children, better mental functioning and less stress). Moreover, these were followed by socio-economic aspects (n = 5) (e.g. increased professional and financial security, better socio-economic position), and behavioural aspects (n = 1) (i.e. improved health behaviour).

#### Disadvantages to the ‘older parent’

In contrast to the advantages, the evidence showed that a majority of disadvantages for both parents were family functioning (n = 4) (e.g. smaller family size, less lifetime with offspring) and psychosocial aspects (n = 1) (fear of social stigma). Additionally, the detected disadvantages for mothers alone (n = 22), regarded mainly psychological aspects (n = 15) (e.g. increased psychological distress, increased risk of depression, increased sense of vulnerability and loss of control, fear of social stigma, fear of loss of independence), and family functioning (n = 7) (e.g. perceived lack of family support, difficulty in transitioning to motherhood). None of the empirical findings of the included studies focused on disadvantages to fathers alone.

## Discussion

In this systematic review, more than two decades of empirical evidence on the psychosocial wellbeing of parents of APA and their offspring have been summarized. Most of the studies had the offspring of APA parents as their main focus, underscoring the attention that is given to the consequences of APA on the children. Much less attention is given to the parents, the group that is at the centre of this decision-making process. The driving motivation of focusing on the children and not the parents apparently was the fear of harming children through parenthood at advanced age. In fact, research shows that although APA is becoming more and more common in many western societies, a significant social stigma of appearing too old to parent exists (Mac Dougall *et al.*, 2012; Nilsen *et al.*, 2012; Woodward and MacDuffie, 2016), even though in some societies, childcare is extensively delegated to grandparents without evidence of serious disadvantages to offspring (Pennings, 2013; Harrison *et al.*, 2017). Also, it is noteworthy that APA parents are often portrayed negatively in the media (Jarvie *et al.*, 2015; Campo-Engelstein *et al.*, 2016), potentially contributing to the existing social stigma. The greater focus on children of APA parents that we found may, therefore, be due to the existing social stigma and additionally to the fact that our review primarily focused on the psychosocial wellbeing in relation to APA, rather than the somatic health consequences to parents of having children later in life. Indeed, other reviews on this topic (Schmidt *et al.*, 2012) have also highlighted the (dis)advantages for mothers of APA in terms, for example, of the increased risk of gestational hypertension, preeclampsia, or gestational diabetes. Also, Lysons and Jadva (2023) focused on the psychosocial outcomes in connection to APA and examined the psychological wellbeing of APA parents with a focus on the parent–child relationship. Their focus, however, pointedly stays in early and middle childhood. At the same time, the results of our review agree with Lysons and Jadva (2023) in that it highlighted how cognitive and behavioural aspects of offspring of APA parents are hard to pinpoint, as the evidence is limited and points towards both advantages in some circumstances and disadvantages in others. Lysons and Jadva (2023) also identify a knowledge gap in how children of APA parents feel about having and being raised by older parents. We agree with their conclusion and wish to add that more research on how parents are doing and how their psychosocial wellbeing develops over time, when they have a child at a later stage in their life, could be helpful to explain the trends in increasing parental age that are widespread in many countries worldwide.

Among studies that primarily focused on the wellbeing of parents, no studies that lay in the scope of this systematic review analysed the fathers exclusively. However, there is some work being carried out with older fathers. For instance, Chaloner (2020) focused on the experience of first-time fathers at 40 years and older. Brandt *et al.* (2019) focused on the association of clinical reproductive risks and advanced paternal age. Couture *et al.* (2021) focused in their review on terminological, social, public health, psychological, ethical, and regulatory aspects when it comes to becoming a father at advanced age. Jadva *et al.* (2022) studied both parents’ psychological health and the experiences in parenthood of older parents who used oocyte donation to conceive. Nevertheless, the fact that we did not find any study on psychological wellbeing and health with an explicit focus on older fathers demonstrates the little importance attributed to older fathers. As a result, there is no empirical evidence on APA fathers. Many studies have highlighted, for example, the increased risks of mental disorders for children whose fathers are older, but none have investigated, for example, whether being a father of advanced age can improve the psychosocial wellbeing of those fathers.

It is also remarkable that only 13% (n = 9) of the studies investigated the effects of APA on the family as a whole. Very little is known about the family structure in APA families and whether they, for example, might flourish and/or overcome (psychosocial) problems of one of their members. From the included studies, only Boivin (2009) addressed parenting competencies, but no other evidence is available about parental competences in the context of APA families. In a study outside of the scope of this systematic review, Trillingsgaard and Sommer (2018) investigated the behaviour of mothers of progressively advanced age towards their children, to see how this develops when both mothers and children get older (measurements on psychosocial functioning were taken of the children at age 7, 11, and 15). Their results suggest that advanced maternal age has some advantages, as they found it is associated with fewer behavioural, social, and emotional difficulties in the mother–child relationship, although only when children were 7 and 11 years old (and not at age 15). Moreover, the authors defined ages >33 (at the time of the birth of the child) as the highest maternal age band, thus making it difficult to draw more specific conclusions specific for mothers having children at a later age (e.g. 40+).

As for the advantages and disadvantages brought to light, the included studies in our systematic review predominantly suggested the existence of disadvantages of APA, specifically in connection to psychopathological and neuro-developmental disorders of children born to older parents. However, we note that other studies bring evidence to light that there are also significant advantages to being an APA parent and a child to APA parents. Also, even though our results point towards (psychosocial) disadvantages dominating the discussion, to conclusively interpret the findings and to form a comprehensible conclusion speaking for or against family building at APA, many more elements have to be considered and investigated. Since it is unlikely that demographic trends in connection to family building (such as the continuously rising parental age trends in many Western societies) are going to drastically change course in the coming years, it is therefore important to not only have more focused research on APA and the consequences to children born into families with parents of advanced age and their parents but also to increase awareness about the involved risks and opportunities in building or enlarging a family at APA. This is especially important in connection to MAR and considerations of (aspiring) parents’ autonomy in decision-making processes (Jindal, 2018). The emphasis on children’s wellbeing which has become evident in our systematic review is an important indicator that, judging from the included articles focusing on the psychosocial wellbeing of involved actors, potentially speaks against parenthood at advanced age. The majority of the disadvantages from the cohort studies included in our review were, for example, the increased risk of schizophrenia as parental age advances (e.g. Wu *et al.*, 2012; Sørensen *et al.*, 2014; Tsuang *et al.*, 2014; Taylor *et al.*, 2019; Weiser *et al.*, 2020; Lan *et al.*, 2021). But, since having a child always entails certain risks, both for the parents and for the child, the question remains of whether the advantages that certain studies indicate (e.g. in terms of higher educational outcomes) can compensate for the higher psychosocial risks. This would be particularly important to clarify, given that studies focusing on APA parents provide evidence concerning socio-economic advantages which could positively affect the overall wellbeing of their children. In any case, many doubts of an ethical nature remain on how to best balance the reproductive freedom of parents and the wellbeing of the child, as highlighted by Pennings (1999).

Our findings further highlight that a distinctive and unified definition of APA is missing, a conclusion that has already been pointed out by Couture *et al.* (2021) and in the case of advanced paternal age by Ramasamy *et al.* (2015). The continued lack of a unified definition is unfortunate for at least three reasons. First, in our review, even though our inclusion criteria aimed at capturing studies on APA where the age of the older parents included was above 40, many studies still used lower thresholds to define APA. This means that the ‘oldest’ category of parents that they analysed may be very broad (e.g. including everyone above 31, 32, or 35), not allowing more specific conclusions to be drawn. Second, there were only a few cases in which a specific and explicit explanation was given to select the boundary beyond which parents were considered of APA in the studies indicated. Thirdly, and relatedly, this leads to a situation in which the most frequent age cut-offs used to define advanced paternal and maternal ages are open for discussion. The age of 35 years often defined older mothers, whereas the age of 40 was used for older fathers. This is curious, given that in many European countries (where most of the studies were located), the average maternal age at birth of the first child is around 30 (Eurostat, 2021), and in countries such as Germany, fathers are aged around 35 years on average when children are born (Destatis, 2020). To acquire more conclusive evidence on the pros and cons of having a child at an APA, one would have to find a common definition and justify why a specific cut-off is used.

Finally, it is noteworthy to underline that most studies were carried out either in Europe, North America, or Asia, leading to a limited geographical picture with a strong focus on highly developed societies. As the demographic shift towards APA is a more prominent theme in such societies and regions, these findings might be expected. However, demographic changes are also taking place or are expected to take place in other countries. Therefore, APA is not a concern for Western societies alone, since, for example, the average age of first-time parenthood has risen by more than three years between 1990 and 2020 in China, reaching 27.5 (Institute of Asia Society Policy, 2023). Also, one very distinct finding of our review is the strong majority of quantitative studies to address psychosocial wellbeing aspects in respect to APA. Moreover, none of the quantitative studies that we identified investigated the many advantages of APA (especially for parents) which have been identified in the qualitative literature. These discrepancies make it even more difficult to provide ‘general’ recommendations (i.e. that take into consideration the overall wellbeing of families involving APA parents) on APA.

As for the limitations of our review, it is important to note that only English-language studies, published between 1 January 2000 and 31 December 2021 found in either PubMed including Medline, Embase, Scopus, PsycInfo, CINAHL, or SocINDEX, based on the search terms that the research team agreed on, were considered for this review. Studies from different databases or in languages other than English or those from other terminologies that we did not include may have, therefore, been missed. For example, in our search, we did not use the term ‘family’ but kept it limited to older parents who would have chosen to build a family at APA. Moreover, the definition of one of our inclusion criteria (the fact that studies had to focus on wellbeing and psychosocial health) was particularly difficult to implement. Our main criterion was to exclude studies that focus exclusively on somatic health (e.g. increased risk of diabetes for mothers or cancer in children). Finally, we included only those studies in which the evidence provided concerned APA parents according to our predefined cut-off of 40 years of age and in which it was evident that at least one of the parents included in the analysis was at least 40 years of age. Unfortunately, this cut-off was not always easy to determine, but this confirms the absence of studies that focus explicitly (and possibly even exclusively) on this group.

## Conclusion

Our results provide a clear picture of the various aspects of offspring’s psychological wellbeing at the centre of the research interest regarding APA parents, and therefore, the interest in having a healthy child with an overall high psychosocial wellbeing. However, significant knowledge gaps remain when it comes to parents and the family as a whole. Virtually no evidence was found on the wellbeing of fathers of advanced age. The obtained results show a gendered view of caregiving, with more interest focused on the wellbeing of the mother as the primary caregiver and virtually no interest in the father as a caregiver. Finally, the definitions of APA varied extensively between studies, precluding comparisons of findings. This lack of comparability between studies complicates discussions surrounding APA and decision-making processes.

Future research should focus on the experiences of offspring of APA parents when they reach an adult age. Of particular interest are long-term family structures of families with APA parents and their psychosocial wellbeing, specifically of the fathers. Also, we propose to invest efforts into additional qualitative research focusing on all aspects of the complex relationship of both APA parents with their children. Finally, studies focusing on families with APA parents in other countries, beyond the Western world, would be greatly informative.

## Supplementary Material

hoad042_Supplementary_Data

## Data Availability

No new data were generated or analysed in support of this research.
