# Supplementary material for: Building a family at advanced parental age: a systematic review on the risks and opportunities for parents and their offspring
Source: Hum Reprod Open. 2023 Nov 15;2023(4):hoad042. doi: 10.1093/hropen/hoad042 (PMC10692762; doi:10.1093/hropen/hoad042)
Supplement: hoad042_Supplementary_Data [file hoad042_supplementary_data.docx]

**Supplementary Table S1. Full search strategy for Pubmed incl. Medline**

| **Population, Context & Outcome** | ((("advanced parental age"[Title/Abstract]) OR ("advanced maternal age"[Title/Abstract]) OR ("advanced paternal age"[Title/Abstract]) OR ("advanced reproductive age"[Title/Abstract]) OR ("late parent*"[Title/Abstract]) OR ("late motherhood"[Title/Abstract]) OR ("late fatherhood"[Title/Abstract])) **AND** (("Fertilization in Vitro"[Mesh]) OR ("Sperm Injections, Intracytoplasmic"[Mesh]) OR ("Reproductive Techniques"[Mesh]) OR ("Reproductive Techniques, Assisted"[Mesh]) OR ("Reproduction"[Mesh]) OR ("Pregnancy"[Mesh]) OR ("IVF"[Title/Abstract]) OR ("in vitro fertilization"[Title/Abstract]) OR ("in-vitro fertilization"[Title/Abstract]) OR ("fertilization in vitro"[Title/Abstract]) OR ("ICSI"[Title/Abstract]) OR ("intracytoplasmic sperm injection"[Title/Abstract]) OR ("reproductive techn*"[Title/Abstract]) OR ("assisted reproductive technolog*"[Title/Abstract]) OR ("assisted reproduction"[Title/Abstract]) OR ("assisted conception"[Title/Abstract]) OR ("reproduction"[Title/Abstract]) OR ("conception"[Title/Abstract]) OR ("birth*"[Title/Abstract]) OR ("pregnan*"[Title/Abstract])) **AND** (("Morals"[Mesh]) OR ("Ethics"[Mesh]) OR ("Social Justice"[Mesh]) OR ("Ageism"[Mesh]) OR ("Risk"[Mesh]) OR ("wellbeing"[Title/Abstract]) OR ("well-being"[Title/Abstract]) OR ("psycho-social"[Title/Abstract]) OR ("social"[Title/Abstract]) OR ("ethical"[Title/Abstract]) OR ("right to reproduce"[Title/Abstract]) OR ("justice"[Title/Abstract]) OR ("family functioning"[Title/Abstract]) OR ("parental competenc*"[Title/Abstract]) OR ("ageism"[Title/Abstract]) OR ("reproductive autonomy"[Title/Abstract]) OR ("outcome*"[Title/Abstract]) OR ("risk*"[Title/Abstract]) OR ("benefit*"[Title/Abstract]))) |
| --- | --- |
| **With the additionally applied filters: “time”, “human” and “language (only English)”** | |

**Supplementary Table S2. Full search strategy for the database Embase**

| **Population, Context & Outcome** | ('advanced maternal age'/exp OR 'advanced parental age':ab,ti OR 'advanced maternal age':ab,ti OR 'advanced paternal age':ab,ti OR 'advanced reproductive age':ab,ti OR 'late parent*':ab,ti OR 'late motherhood':ab,ti OR 'late fatherhood':ab,ti) **AND** ('ivf'/exp OR 'in vitro fertilization'/exp OR 'intracytoplasmic sperm injection'/exp OR 'reproductive technologies'/exp OR 'assisted reproduction'/exp OR 'assisted conception'/exp OR 'reproduction'/exp OR 'conception'/exp OR 'birth'/exp OR 'pregnancy'/exp OR 'ivf':ab,ti OR 'in vitro fertilization':ab,ti OR 'in-vitro fertilization':ab,ti OR 'fertilization in vitro':ab,ti OR 'icsi':ab,ti OR 'intracytoplasmic sperm injection':ab,ti OR 'reproductive techn*':ab,ti OR 'assisted reproductive technolog*':ab,ti OR 'assisted reproduction':ab,ti OR 'assisted conception':ab,ti OR 'reproduction':ab,ti OR 'conception':ab,ti OR 'birth*':ab,ti OR 'pregnan*':ab,ti) **AND** ('wellbeing'/exp OR 'social'/exp OR 'justice'/exp OR 'family functioning'/exp OR 'ageism'/exp OR 'outcome'/exp OR 'risk'/exp OR 'wellbeing':ab,ti OR 'well-being':ab,ti OR 'psycho-social':ab,ti OR 'social':ab,ti OR 'ethical':ab,ti OR 'right to reproduce':ab,ti OR 'justice':ab,ti OR 'family functioning':ab,ti OR 'parental competenc*':ab,ti OR 'ageism':ab,ti OR 'reproductive autonomy':ab,ti OR 'outcome*':ab,ti OR 'risk*':ab,ti OR 'benefit*':ab,ti) |
| --- | --- |
| **With the additionally applied filters: “time”, “human” and “language (only English)” and excluding conference abstracts, conference papers and conference reviews.** | |

**Supplementary Table S3. Full search strategy for the database Scopus**

| **Population, Context & Outcome** | ( TITLE-ABS-KEY ( ( "advanced parental age" ) OR ( "advanced maternal age" ) OR ( "advanced paternal age" ) OR ( "advanced reproductive age" ) OR ( "late parent*" ) OR ( "late motherhood" ) OR ( "late fatherhood" ) ) AND TITLE-ABS-KEY ( ( "IVF" ) OR ( "in vitro fertilization" ) OR ( "in-vitro fertilization" ) OR ( "fertilization in vitro" ) OR ( "ICSI" ) OR ( "intracytoplasmic sperm injection" ) OR ( "reproductive techn*" ) OR ( "assisted reproductive technolog*" ) OR ( "assisted reproduction" ) OR ( "assisted conception" ) OR ( "reproduction" ) OR ( "conception" ) OR ( "birth*" ) OR ( "pregnan*" ) ) AND TITLE-ABS-KEY ( ( "wellbeing" ) OR ( "well-being" ) OR ( "psycho-social" ) OR ( "social" ) OR ( "ethical" ) OR ( "right to reproduce" ) OR ( "justice" ) OR ( "family functioning" ) OR ( "parental competenc*" ) OR ( "ageism" ) OR ( "reproductive autonomy" ) OR ( "outcome*" ) OR ( "risk*" ) OR ( "benefit*" ) ) ) |
| --- | --- |
| **With the additionally applied filters: “time”, “human” and “language (only English)” and excluding conference papers.** | |

**Supplementary Table S4. Full search strategy for the database PsycInfo**

| **Population** | (advanced parental age OR advanced maternal age OR advanced paternal age OR advanced reproductive age OR late parent* OR late motherhood OR late fatherhood).m_titl. or  (advanced parental age OR advanced maternal age OR advanced paternal age OR advanced reproductive age OR late parent* OR late motherhood OR late fatherhood).ab. |
| --- | --- |
| **Context** | (exp Reproductive Technology/ or exp Birth/ or exp Pregnancy) or  (ivf or in vitro fertilization or in-vitro fertilization or fertilization in vitro or ICSI or intracytoplasmic sperm injection or reproductive techn* or assisted reproductive technolog* or assisted reproduction or assisted conception or reproduction or conception or birth* or pregnan*).m_titl. or  (ivf or in vitro fertilization or in-vitro fertilization or fertilization in vitro or ICSI or intracytoplasmic sperm injection or reproductive techn* or assisted reproductive technolog* or assisted reproduction or assisted conception or reproduction or conception or birth* or pregnan*).ab. |
| **Outcome** | (exp Well Being/ or exp Psychological Factors/ or exp Ethics/ or exp Justice/ or exp Ageism/) or  (wellbeing or well-being or psycho-social or social or ethical or right to reproduce or justice or family functioning or parental competenc* or ageism or reproductive autonomy or outcome* or risk* or benefit*).m_titl. or  (wellbeing or well-being or psycho-social or social or ethical or right to reproduce or justice or family functioning or parental competenc* or ageism or reproductive autonomy or outcome* or risk* or benefit*).ab |
| **With the additionally applied filters: “time”, “human” and “language (only English)”.** | |

**Supplementary Table S5. Full search strategy for the database CINAHL**

| **Population** | TI ((“advanced parental age”) OR (“advanced maternal age”) OR (“advanced paternal age”) OR (“advanced reproductive age”) OR  (“late parent*”) OR (“late motherhood”) OR (“late fatherhood”)) OR  AB ((“advanced parental age”) OR (“advanced maternal age”) OR (“advanced paternal age”) OR (“advanced reproductive age”) OR  (“late parent*”) OR (“late motherhood”) OR (“late fatherhood”)) |
| --- | --- |
| **Context** | ((MM “Fertilization in Vitro”) OR (MM “Reproduction Techniques”) OR (MM “Reproduction”) OR (MM “Pregnancy”)) OR  TI ((“IVF”) OR (“in vitro fertilization”) OR (“in-vitro fertilization”) OR (“fertilization in vitro”) OR (“ICSI”) OR (“intracytoplasmic sperm injection”) OR (“reproductive techn*”) OR (“assisted reproductive technolog*”) OR (“assisted reproduction”) OR (“assisted conception”) OR (“reproduction”) OR (“conception”) OR (“birth*”) OR (“pregnan*”)) OR  AB ((“IVF”) OR (“in vitro fertilization”) OR (“in-vitro fertilization”) OR (“fertilization in vitro”) OR (“ICSI”) OR (“intracytoplasmic sperm injection”) OR (“reproductive techn*”) OR (“assisted reproductive technolog*”) OR (“assisted reproduction”) OR (“assisted conception”) OR (“reproduction”) OR (“conception”) OR (“birth*”) OR (“pregnan*”)) |
| **Outcome** | ((MM “Ethics”) OR (MM “Family Functioning”) OR (MM “Ageism”)) OR  TI ((“wellbeing”) OR (“well-being”) OR (“psycho-social”) OR (“social”) OR (“ethical”) OR (“right to reproduce”) OR (“justice”) OR (“family functioning”) OR (“parental competenc*”) OR (“ageism”) OR (“reproductive autonomy”) OR (“outcome*”) OR (“risk*”) OR (“benefit*”)) OR  AB ((“wellbeing”) OR (“well-being”) OR (“psycho-social”) OR (“social”) OR (“ethical”) OR (“right to reproduce”) OR (“justice”) OR (“family functioning”) OR (“parental competenc*”) OR (“ageism”) OR (“reproductive autonomy”) OR (“outcome*”) OR (“risk*”) OR (“benefit*”)) |
| **With the additionally applied filters: “time”, “human” and “language (only English)”.** | |

**Supplementary Table S6. Full search strategy for the database SocINDEX**

| **Population** | TI ((“advanced parental age”) OR (“advanced maternal age”) OR (“advanced paternal age”) OR (“advanced reproductive age”) OR (“late parent*”) OR (“late motherhood”) OR (“late fatherhood”)) OR  AB ((“advanced parental age”) OR (“advanced maternal age”) OR (“advanced paternal age”) OR (“advanced reproductive age”) OR (“late parent*”) OR (“late motherhood”) OR (“late fatherhood”)) |
| --- | --- |
| **Context** | ((DE “fertilization in vitro”) OR (DE “reproductive technology”) OR (DE “reproduction”) OR (DE “pregnancy”)) OR  TI ((“IVF”) OR (“in vitro fertilization”) OR (“in-vitro fertilization”) OR (“fertilization in vitro”) OR (“ICSI”) OR (“intracytoplasmic sperm injection”) OR (“reproductive techn*”) OR (“assisted reproductive technolog*”) OR (“assisted reproduction”) OR (“assisted conception”) OR (“reproduction”) OR (“conception”) OR (“birth*”) OR (“pregnan*”)) OR  AB ((“IVF”) OR (“in vitro fertilization”) OR (“in-vitro fertilization”) OR (“fertilization in vitro”) OR (“ICSI”) OR (“intracytoplasmic sperm injection”) OR (“reproductive techn*”) OR (“assisted reproductive technolog*”) OR (“assisted reproduction”) OR (“assisted conception”) OR (“reproduction”) OR (“conception”) OR (“birth*”) OR (“pregnan*”)) |
| **Outcome** | ((DE “well-being”) OR (DE “psychosocial factors”) OR (DE “ethics”) OR (DE “ageism”)) OR  TI ((“wellbeing”) OR (“well-being”) OR (“psycho-social”) OR (“social”) OR (“ethical”) OR (“right to reproduce”) OR (“justice”) OR (“family functioning”) OR (“parental competenc*”) OR (“ageism”) OR (“reproductive autonomy”) OR (“outcome*”) OR (“risk*”) OR (“benefit*”)) OR  AB ((“wellbeing”) OR (“well-being”) OR (“psycho-social”) OR (“social”) OR (“ethical”) OR (“right to reproduce”) OR (“justice”) OR (“family functioning”) OR (“parental competenc*”) OR (“ageism”) OR (“reproductive autonomy”) OR (“outcome*”) OR (“risk*”) OR (“benefit*”)) |
| **With the additionally applied filters: “time” and “language (only English)”.** | |
